# New solutions to capture and enrich bacteria from complex samples

**DOI:** 10.1007/s00430-020-00659-1

**Published:** 2020-02-05

**Authors:** Maria G. Sande, Tugçe Çaykara, Carla Joana Silva, Ligia R. Rodrigues

**Affiliations:** 1 CEB-Centre of Biological Engineering, Universidade do Minho, Campus de Gualtar, 4710-057 Braga, Portugal; 2 CENTI-Center for Nanotechnology and Smart Materials, Rua Fernando Mesquita 278, 4760-034 Vila Nova de Famalicão, Portugal

**Keywords:** Diagnostics, Bacteria capture, Enrichment, Detection limit, Microfluidics, Magnetic beads, Magnetic nanoparticles, Acoustophoresis, Aptamer, Bacteriophage, Peptide, Point-of-care, Nano-patterning

## Abstract

Current solutions to diagnose bacterial infections though reliable are often time-consuming, laborious and need a specific laboratory setting. There is an unmet need for bedside accurate diagnosis of infectious diseases with a short turnaround time. Moreover, low-cost diagnostics will greatly benefit regions with poor resources. Immunoassays and molecular techniques have been used to develop highly sensitive diagnosis solutions but retaining many of the abovementioned limitations. The detection of bacteria in a biological sample can be enhanced by a previous step of capture and enrichment. This will ease the following process enabling a more sensitive detection and increasing the possibility of a conclusive identification in the downstream diagnosis. This review explores the latest developments regarding the initial steps of capture and enrichment of bacteria from complex samples with the ultimate goal of designing low cost and reliable diagnostics for bacterial infections. Some solutions use specific ligands tethered to magnetic constructs for separation under magnetic fields, microfluidic platforms and engineered nano-patterned surfaces to trap bacteria. Bulk acoustics, advection and nano-filters comprise some of the most innovative solutions for bacteria enrichment.

## Introduction

Infectious diseases including lower respiratory infections, tuberculosis and diarrheal diseases are among the top ten causes of mortality globally, ahead of dementia, cancer and diabetes mellitus [1]. For instance, every year, nearly 1.7 billion cases of childhood diarrheal disease are registered, being mainly transmitted by pathogens such as *Escherichia coli* present in contaminated food and water [2]. The scale of diseases prevalence, multiplicity of bacterial species and strains involved and disease incidence, in addition to the emergence of opportunistic pathogens, make clinical diagnostics a challenging task, especially in places with lack of resources.

Culture-based assays continue to be the gold standard for bacteria identification [3]. However, this methodology is time-consuming taking several days in some cases [4]. Moreover, highly skilled labor and a specific laboratory setting is required, which can limit their use in some places where proper facilities are not available. In the last decades, several immunological and molecular methods have been developed for pathogen diagnostics with a high level of sensitivity and accuracy. The most popular methods include immuno-based biosensors, enzyme-linked immunosorbent assay (ELISA) and molecular methods such as polymerase chain reaction (PCR), mass spectrometry (MS) and more recently, loop-mediated isothermal amplification (LAMP) and next generation sequencing (NGS) [4]. Though highly sensitive, these methods involve complex, time-consuming sample preparation steps, sophisticated laboratory equipment and highly skilled labor.

**Fig. 1:**
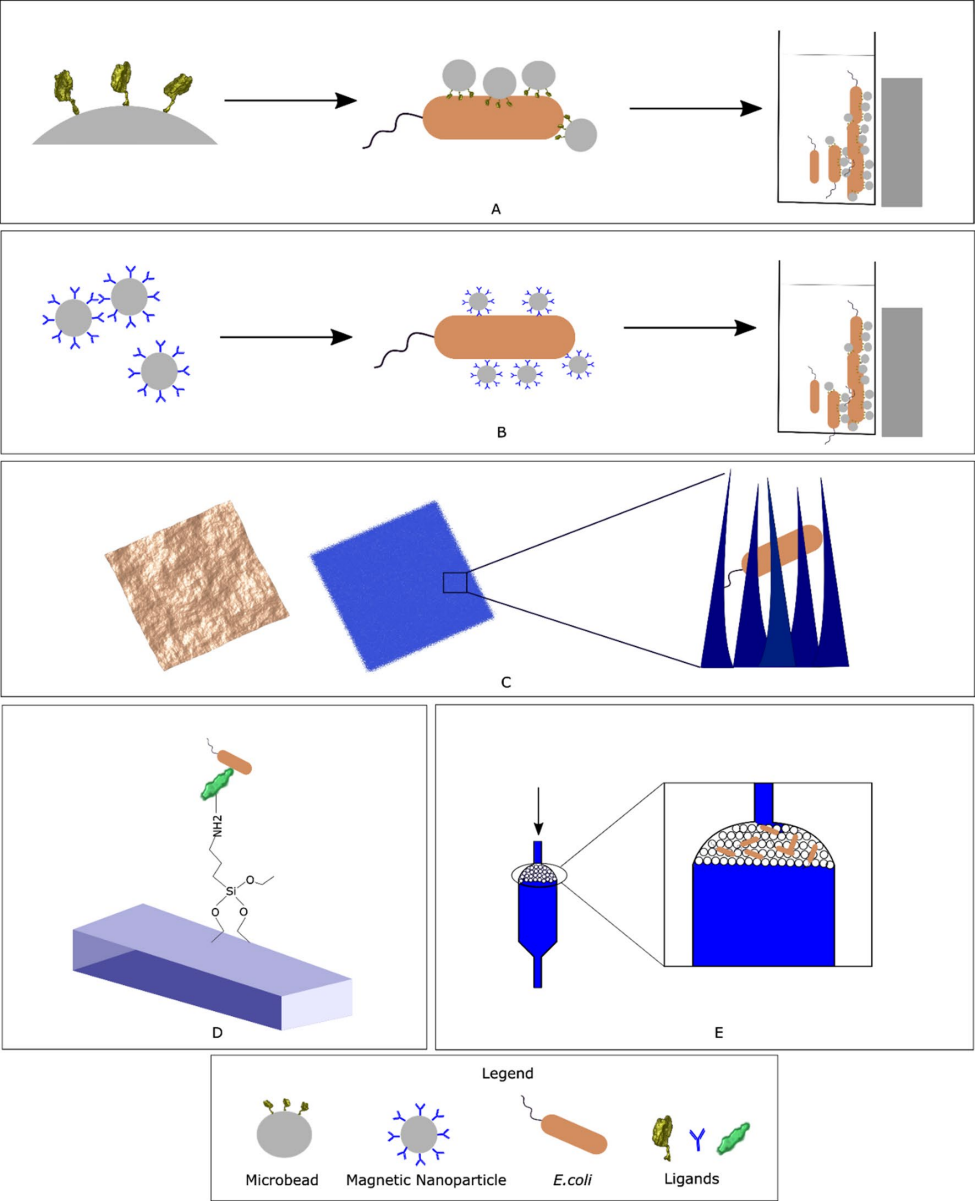
Overview of the methods to capture bacteria from biological samples. **a** Magnetic beads functionalized with ligands that bind bacteria and can be enriched by magnetic separation. **b** Functionalized magnetic nanoparticles are used to bind bacteria and are enriched by magnetic separation. **c** Various types of nano-topographies such as prickly or nano-patterned surfaces can be engineered to trap bacteria. **d**. Surfaces functionalized with chemical cross linkers and ligands that can directly capture bacteria. **e** Physical barriers such as nano-filters can be used to trap bacteria from a sample flowing through it. Combinations of all these methods have also been explored to capture bacteria from complex samples

Despite of the current availability of a multitude of bacteria identification methods, there is still an enormous unmet need for fast, easy to use, cost-effective and highly sensitive point-of-care (POC) diagnostics. Capture and enrichment techniques can be achieved with compact devices, which can potentially provide bedside diagnostics. In addition, sometimes the number of pathogens present in biological samples are lower than the detection limits of the available methods, thus requiring additional pre-enrichment steps which add costs to the diagnostics and increase the turnaround time. Moreover, samples usually associated with low bacterial numbers (e.g., CNS fluid) or in mixed populations (e.g., stool), can benefit from a diagnostic approach using capture and enrichment of samples prior to downstream processing. This review explores the most recent developments regarding materials, methods and approaches for capturing and/or enriching bacteria from biological complex samples towards the design of more straight-forward and cost-effective clinical diagnostic methods to be used in a POC setting (Fig. 1).

### New materials and methods to capture and enrich bacteria from biological samples

Many efforts have been conducted in the recent years towards the development of innovative diagnostics against several diseases. These involve the search and engineering of specific ligands (antibodies, peptides, aptamers), the design and fabrication of new materials exhibiting a panoply of relevant physicochemical features (hydrophilicity/hydrophobicity; conductivity; wettability; rigidity; roughness; flexibility; biocompatibility; among others), and the combination of different separation (affinity, molecular weight, acoustic) and detection (fluorescence, electrochemistry, absorbance) principles to enable the efficient capture of specific pathogens from complex samples (blood, urine, stool), their enrichment and qualitative and/or quantitative detection.

### Magnetic beads

Aptamers can be raised against virtually any target through SELEX (Systematic evolution of ligands by exponential enrichment) [7], thus opening a number of opportunities for the development of innovative and cheap diagnostics. A POC, aptamer-mediated biosensor was developed to aid in malaria diagnosis [5]. Aptamers are oligonucleotides fragments (DNA or RNA) that bind to their targets with high affinity and specificity [6]. An aptamer that specifically recognizes the *Plasmodium falciparum* lactate dehydrogenase (PfLDH) enzyme was designed and coated onto magnetic microbeads towards its magnet-guided capture and colorimetric detection on a microfluidic platform. Moreover, the biosensor developed a signal within 20 min of adding a development reagent and the cost of the microfluidic platforms are not expensive costing about 3 USD per cassette.

In a recent work [8], the long tail fibers (S16 LTF) of a bacteriophage that specifically binds *Salmonella* were immobilized onto paramagnetic beads that were further used to capture *Salmonella* cells from different samples. Bacteriophages are viruses that can only live and replicate within bacteria. They infect hosts by binding to surface receptors by their long tail fibers with unmatched specificity. Due to the inherent specificity of these bacteriophage tail fiber to bind distinct bacterial species they are suitable affinity molecules. Concentrations of captured bacteria between 10^2^ and 10^7^ CFU/mL were detected by a change in color of the enzymatic label used. Alternatively, specific peptides against different target pathogens can be selected by Phage Display [7] and further used to modify magnetic beads leading to new diagnostics.

Most reports provide proof-of-concept studies using synthetic samples to validate their capture and enrichment models. However, the results do not always translate to a scenario where real samples are used as discussed by Bicart-See and collaborators [9]. These authors evaluated the success of bacteria capture from clinical samples. *Staphylococcus aureus* was isolated from articular fluid and synovial tissue using magnetic beads coated with an engineered chimeric human opsonin protein, Fc-mannose-binding lectin (FcMBL). The direct capture from clinical samples was found to be very inefficient (lower than 5%). Masking of FcMBL binding sites by immunoglobulins and immune cells, as well as the high viscosity of complex biological samples were found to be the main reason responsible for the low capture efficiency. However, through a pre-treatment of the clinical tissue samples with hypotonic washes, hyaluronidase and proteases, FcMBL capture efficiency increased to 76%.

### Magnetic nanoparticles

Wang and colleagues [10] developed a technique using iron oxide magnetic nanoparticles (MNP) functionalized with aptamers to specifically bind *S. aureus* and *E. coli*. Additional functionalization groups involved a photosensitizer chlorin e6 for detection by fluorescence microscopy and polyethylene glycol (PEG) to improve the stability and biocompatibility of the MNPs. The ultimate goal was to provide a quick turnaround time for diagnosis of sepsis, which can be fatal if not reversed within few hours. The developed solution enabled capturing and detecting trace amounts of bacteria (10 CFU) within 1.5 h from mouse blood.

Similarly, MNPs containing an iron oxide magnetite core were functionalized with an amino-glycan coating [11]. These MNPs were fixed to plastic strips and used to rapidly capture bacteria directly from complex matrices by dipping the strips into the sample. The MNP fixed to the strips have hydroxyl, amino and hydrophobic regions that interact with bacterial membranes and their surface epitopes forming non-covalent electrostatic bonds similar to those found in antibody bonding. Overall, the MNP-strip capture aided with a cyclic voltammetry detection system, detected cells in less than 30 min, without requiring an initial separation of targeted cells from the matrix. In addition, the use of plastic strips for bacteria capture significantly reduces costs.

In a recent development [12], a microfluidic channel was designed with a slanted ridge array that induces advective spiral flows when a sample fluid flows through the microfluidic channel causing enhanced separation of magnetized particles present in the sample. Cells bound with magnetic particles in the presence of the advective flow are repeatedly transferred close to a ferromagnetic nickel structure, where they can be captured easily, since the magnetic field is the strongest in the vicinity of the magnet. This system was validated using super-paramagnetic nanoparticles coated with mannose-binding lectin and bound to *E. coli* spiked onto undiluted whole blood from rats. 91.6% of *E. coli* bound to the MNPs were successfully separated as compared to only 23.9% of *E. coli* collected in a conventional microfluidic device.

### Nano-patterned 3D surfaces

Nano-patterned 3D surfaces have higher binding capacities compared with flat substrates [13]. These surfaces offer more reactive sites to bind with bacterial targets. Several nanostructures have been evaluated regarding their ability to capture the target bacteria, including nanowires, nanofractals and nanofibers [14].

Zinc oxide nanorod arrays were fabricated by an electrochemical method to form a 3D nano–bio surface functionalized with lectin Concanavalin A (ConA) to capture *E. coli* [15]. Lectins are effective ligands due to their specificity of binding polysaccharide components on bacterial cells surfaces. The results showed a reasonable detection limit of 0.9 × 10^2^ CFU/mL in saline-spiked *E. coli.*

Li and colleagues [14] further demonstrated the synergistic effect of 3D topography coupled with surface chemical modification to increase bacteria attachment and binding efficiency. Densely packed nanowire arrays were fabricated on silicon wafers such that the space between nanowire structures was smaller than the diameter of a bacterium to improve the binding. ConA molecules were introduced onto the nanowires through sequential chemical covalent coupling to confer selective bacteria recognition capabilities. ConA has high affinity for bacterial lipopolysaccharide components and the capture sensitivity of test assays was 10 CFU/mL. The functionalized nanowires were found to capture up to ten times more *S. aureus* than the silicon‐ConA substrate without nanowires and within a short incubation time of 30 min, thus suggesting the critical role of the 3D topography in an enhanced capture of bacteria.

A dialyzer made with 3D carbon foam and grafted with nanowires was used to capture bacteria from human blood samples [16]. Pointed polycrystalline nanowires bent readily to form 3D ‘nanoclaws’ to trap bacteria due to a decreasing Young’s moduli from the bottom to the tip resulting from sample flow. Bacteria-capture efficiency was improved from 10% on plain carbon foam and 40% on unbendable nanowires on carbon foam to 97% on bendable polycrystalline nanowires on carbon foam when placed in a simulated bloodstream flowing at 10 cm/s.

Sensors were fabricated by in situ deposition of prickly Zn–CuO nanoparticles and graphene oxide nanosheets on an Ni porous electrode [17]. Due to burr-like nanostructures on the surface, the sensor exhibited excellent bacteria-capture efficiency (70–80% in 20 min) at low concentrations of 50 CFU/mL, and high detection sensitivity with lower limit of 10 CFU/mL. An excellent detection of bacteria at concentrations ranging from 10 to 10^5^ CFU/mL within a short time interval of 30 min was observed using an *E. coli* spiked rat blood sample.

### Methods involving specific chemicals and ligands

Wang et al. [18] developed a multifunctional chip comprising a silicon wafer functionalized with 4-mercaptophenylboronic (4-MPBA). The 4-MPBA molecule possesses a boronic acid group that is known to reversibly bind peptidoglycan from bacterial cell walls enabling the specific capture of several bacteria. In addition, 4-MPBA has also been bound to silver nanoparticles for cell lysis and to a benzene ring that can amplify signals of the captured bacteria for their detection and identification. In terms of efficiency, 1 × 10^2^ cells/mL of *E. coli* or *S. aureus* were accurately identified in human blood, thus making it sensitive enough to detect bacteria in the blood of sepsis patients. Yang et al. [19] reported a similar solution using 4-MPBA on a semiconductor substrate. The platform was able to capture *E. coli* with high specificity and sensitivity with a detection limit as low as 46 CFU/mL of *E. coli* from a test suspension. Moreover, this class of SERS chip can be easily and cheaply fabricated increasing the likelihood of its use for practical applications.

A herringbone-type microfluidic platform contained a Magainin 1 peptide immobilized on its surface to enrich a test bacteria from urine samples [20]. Magainin 1 semi-selectively binds to Gram-negative bacteria species. The platform was coupled with a recombinase polymerase amplification (RPA) sensor for simultaneous pathogenic DNA amplification and detection in a real time and highly sensitive manner. The detection limit was found to be 20 times more sensitive in 10 mL urine with *Salmonella* and ten times more sensitive in 10 mL urine with *Brucella* than that of real-time PCR without the enrichment step. The combination of ligand-based enrichment and an RPA sensor exhibited a detection sensitivity of 5 CFU/mL urine for *Salmonella* and 10 CFU/ml urine for *Brucella* within 60 min.

### Methods involving sound wave acoustophoresis

A number of microfluidic separation methods have been used to separate bacteria from the bulk sample such as centrifugal microfluidics and dielectrophoresis [21]. Acoustophoresis has recently been demonstrated as an effective technique to separate components from a bulk fluid in the presence of sound waves into separate streams [21]. This method has proven to be useful in sepsis diagnosis due to the quick turnaround time and possibly for blood disinfection given that it can be scaled-up to handle larger volumes.

In a recent example [22], bulk acoustophoresis was used to separate bacteria from the blood cells from whole blood samples. The small size of bacteria prevents them from being affected by an acoustic field, and therefore, they remain in the blood plasma flow that is directed to a separate outlet. Upon optimization of the sample volume, it was found that 1 ml of undiluted whole blood can be processed within 12.5 min, with a bacteria recovery of 90% and almost 100% blood cell removal.

In addition, bulk acoustophoresis was used to identify bacteria using a bacteriophage-based luminescence assay in a microfluidic chip [23]. To demonstrate the effectiveness of the acoustophoretic system, three clinically relevant species of bacteria were tested, namely, *Pseudomonas aeruginosa, S. aureus and E. coli.* Bacteria recovery from blood samples was between 45% and 60% across species while removing more than 85% of blood cells.

### Methods involving physical barriers

Novel microfluidic immune-affinity columns consisting of packed microbeads functionalized with antibodies were used to provide large surface areas for effective capture of uropathogenic *E. coli* [24]. Due to the capillary action of the channels in the columns, the sample, antibody and fluorescent label were dispensed autonomously and the assay time was very fast (7 min). The detection limit of *E. coli* in synthetic urine was 1.2 × 10^2^ CFU/mL, which is well below the cutoff of 10^5^ CFU/mL of a single organism which is often used as a cutoff point in the diagnosis of uncomplicated urinary tract infections.

Another device was fabricated based on the self-assembly of polystyrene microparticles at a pillar array region within a microfluidic chip to form a nanoscale filter for bacteria capture from samples flowing on gold nano/micro islands [25]. The nanosurface microfluidic device showed an average capture efficiency of 93% *E. coli* from samples.

### Methods involving mixed separation principles

PEG hydrogel bead-like scaffolds were functionalized with aptamers to specifically target *S. aureus* and *E. coli*. PEG is used to decrease non-specific adsorption of other targets. This PEG scaffold was tagged with MNPs to simplify the processing of the bioassays. In addition, the scaffolds were nano-patterned to increase the surface area for probe immobilization and greatly enhance binding efficiency of target bacteria. Multiple bacterial species were captured from human blood spiked with *S. aureus* and *E. coli* with concentrations as low as 100 CFU/mL within 2.5 h [26].

A reusable supramolecular platform was developed for the specific capture and release of bacteria and proteins [27]. The platform exploited the carbohydrate–protein affinity recognition to capture specific proteins and bacteria. It used multi-layering polyelectrolyte films, followed by the incorporation of β-cyclodextrin (β-CD) derivatives modified with mannose as host molecules which were demonstrated to bind type I fimbriae *E. coli* and lectin proteins. The special feature of this platform is that captured bacteria could be released from the surface by incubation with sodium dodecyl sulfate and the platform can then be reused. This versatile and reusable system is very promising and could comprise the basis for the development of new, cheap and reusable diagnostic devices.

## Conclusion and outlook

Classical methods used for bacterial infection diagnosis are reliable, however, in an era where faster, more convenient and flexible solutions are constantly demanded, the realm of diagnostics is no exception. There is a clear need for faster results using simple and cheap miniaturized platforms. Innovators in the diagnostics field have been inspired by the semiconductor industry to develop microfluidic devices and ‘chips’ to capture, detect and quantify diverse microorganisms. The great advantage of these microfluidic devices and compact sensors is that they are cheaply manufactured, costing as little as a few USD in many cases reducing the overall costs. Other cost-saving examples include the use of materials such as polystyrene and paper to manufacture some of the innovative devices herein described, or the use of aptamers as specific ligands, which are cheap and easy to synthesize.

In addition, relevant contributions from the nanotechnology and synthetic biology fields led to the development of targeted micro/nanoparticles for capture and detection purposes. Immunoassays, chemical ligand-mediated capture and functionalized magnetic microbeads or nanoparticles are among the successful solutions developed to capture bacterial cells in microfluidic devices. As a result, turnaround times have been reduced from many hours to minutes in some cases and miniaturization promises fast, sensitive and easy to use POC diagnostic devices. However, before these new solutions translate into an important improvement in the infection diagnostics field, they must be tested beyond the developmental stage. One relevant challenge includes validating these new solutions using patient samples (blood, urine, stool) that are more complex and heterogeneous than the synthetic samples commonly used at the developmental stage. Therefore, it is always important to bear in mind that even if a diagnostic method performs very well with synthetic samples, it might need further adjustments and optimizations before it can be used in a real context with biological samples. In addition, while in theory, these new solutions appear to be cost-effective, some use expensive ligands (e.g., antibodies) and, for instance, the design of complex and sophisticated microfluidic platforms can also significantly increase costs. Other solutions being proposed bypass the analyte–ligand binding making use of nano-patterned surfaces or micro/nano-obstacles and filters to capture bacteria. However, these solutions may lack specificity. An alternative approach could be to use inexpensive ligand biomolecules (e.g., aptamers) immobilized onto available and common polymeric substrates (e.g., silicon), thereby taking advantage of the specificity and sensitivity of the ligand coupled to a cheap platform. The development of these innovative solutions will undoubtedly lead to a new generation of tools to capture and detect bacteria, thus making POC diagnosis of an infection within hours or even minutes and overcoming the limitations of the conventional methods, a reality.
